# Supplementary material for: Using text data instead of SIC codes to tag innovative firms and classify industrial activities
Source: PLoS One. 2022 Jun 30;17(6):e0270041. doi: 10.1371/journal.pone.0270041 (PMC9246165; doi:10.1371/journal.pone.0270041)
Supplement: Attachment — Submitted filename: Response to reviewers REV.docx [file pone.0270041.s001.docx]

**Response to reviewers**

First, we would like to thank the referees for useful comments and the Editor for the chance to publish on PLOS ONE.

Through this note we intend to provide the Editor and the referees with a detailed list of the revisions brought to the manuscript. We considered all the *editor and referees’ comments (in italic and blue ink)*:

- replies and short comments are in black ink
- revised text is in green ink

At the end of the process of revision, our paper was substantially improved in several respects. Thank you for your time and feedback.

The editor and referees’ suggestions have been collected and organized according to the order followed in the mail sent to the authors (PLOS ONE Decision: Revision required [PONE-D-21-29097] - [EMID:36dfc19c49cd2b78]). There are three sections:

- A) EDITOR
- B) REVIEWERS’ MAJOR POINTS
- C) REVIEWERS’ MINOR POINTS

A) EDITOR

*# 1 EDITOR: Overall, while there is a contributing point in the paper, there is mismatch between the current literature review, title, discussion and contributing area. The title should be revised as well. I recommend authors to align well the introduction, literature review, analysis and conclusion.*

Ok, revised

The emphasis has been placed on the contribution of the paper, which uses text mining and semantic algorithms to tag innovative firms and offer an alternative perspective to classify industrial activities. Evidence is interesting because allows us to understand ‘what firms do’ in a more penetrating and updated way than referring to SIC codes.

The title has been revised. The introduction and literature review have been aligned to the analysis and the conclusions.

Considering the suggestions by reviewers, we have given emphasis to the activities of tagging and classifying.

However, as suggested by one reviewer, we opted to relegate the issue of proximity between firms as a further and complementary issue, in addition to the above focus of the paper. In effect, through matching firms’ keywords and industrial classifications, we are able explore the degree of interconnection between the firms under observation, that is a measure by which researchers can derive industrial proximity.

This is the new revised (long) title:

Using text data (instead of SIC codes) to tag innovative firms and classify industrial activities

The proposed short title is:

Using text data instead of SIC codes to classify firms

This is the revised abstract based on reviewers’ suggestions:

The paper uses text mining and semantic algorithms to tag innovative firms and offer an alternative perspective to classify industrial activities. Instead of referring to firms’ standard industrial classification codes, we gather information from companies’ websites and corporate purposes, extract keywords and generate tags concerning firms’ activities, specializations, and competences. Evidence is interesting because allows us to understand ‘what firms do’ in a more penetrating and updated way than referring to standard industrial classification codes. Moreover, through matching firms’ keywords, we can explore the degree of closeness between the firms under observation, a measure by which researchers can derive industrial proximity. The analysis can provide policymakers with a detailed and comprehensive picture of the innovative trajectories underlying the industrial structure in a geographic area.

*# 2 EDITOR: If you would like to make changes to your financial disclosure, please include your updated statement in your cover letter.*

Ok, revised.

For this point we refer to the Funding Statement. Please see point # 6 EDITOR below.

*# 3 EDITOR: Guidelines for resubmitting your figure files are available below the reviewer comments at the end of this letter.*

Ok, revised.

Figure files have been uploaded separately.

*\# 4 EDITOR: If applicable, we recommend that you deposit your laboratory protocols in* [*protocols.io*](http://protocols.io) *to enhance the reproducibility of your results.*

Ok, revised. See point # 2 REVIEWER below.

*# 5 EDITOR: 1. Please ensure that your manuscript meets PLOS ONE's style requirements, including those for file naming.*

Ok, revised.

The manuscript has been modified to meet the PLOS ONE’s style requirements available at <https://journals.plos.org/plosone/s/submission-guidelines#loc-manuscript-organization> .

*# 6 EDITOR: 2. Please remove any funding-related text from the manuscript and let us know how you would like to update your Funding Statement. Please include your amended statements within your cover letter; we will change the online submission form on your behalf.*

Ok, revised.

Below the Funding Statement:

Fondirigenti G. Taliercio (Interprofessional fund for continuous training of managers promoted by Confindustria and Federmanager; www.fondirigenti.it) provided funding for the Strategic Initiative ‘Modelling of an Observatory to monitor the innovation ecosystem in the Chieti and Pescara area and mapping of managerial skills with a high innovation rate’, grant number: CIG 8188368708. The funder had no role in study design, data collection and analysis, decision to publish, or preparation of the manuscript. The specific roles of the authors AM and CB are articulated in the ‘author contribution’ section.

*# 7 EDITOR: 3. Please complete your Competing Interests on the online submission form to state any Competing Interests. This information should be included in your cover letter; we will change the online submission form on your behalf.*

Ok, revised.

Below the statement on Competing Interests:

The authors AM and CB founded the academic spinoff Explo. Explo is specialized on text analytics. This does not alter our adherence to PLOS ONE policies on sharing materials and data. Explo does not have any patent, patent application, or product in development or for market related to the research article.

*# 8 EDITOR: 4. We note that you have stated that you will provide repository information for your data at acceptance. Should your manuscript be accepted for publication, we will hold it until you provide the relevant accession numbers or DOIs necessary to access your data. If you wish to make changes to your Data Availability statement, please describe these changes in your cover letter and we will update your Data Availability statement to reflect the information you provide.*

Ok, revised.

Below the statement on Data Availability:

The data underlying this research article comes from different databases: startup.registroimprese.it by Unioncamere is the official database of the Chambers of Commerce that collects Italian startups and innovative SMEs and provides upon request free lists of registered companies; AIDA (Analisi Informatizzata delle Aziende) by Bureau Van Dijk includes comprehensive information concerning corporate purposes and financial indicators on companies in Italy. AIDA is a commercial database and data are accessible, after registration, at the following link: <https://aida.bvdinfo.com>. We made the minimal data set related to the values used to build analysis and graphs publicly available without restrictions on the web repository GitHub, accessible at the following link: <https://github.com/cbaldassari/2022-plosone>.

*# 9 EDITOR: 5. We note that [Figure 1] in your submission contain [map/satellite] images which may be copyrighted. All PLOS content is published under the Creative Commons Attribution License (CC BY 4.0), We require you to either (1) present written permission from the copyright holder to publish these figures specifically under the CC BY 4.0 license, or (2) remove the figures from your submission:*

*a. You may seek permission from the original copyright holder of Figure 1 to publish the content specifically under the CC BY 4.0 license.*

Ok, revised.

Figure 1 has been generated via OpenStreetMap. OpenStreetMap is an open-source collaborative mapping project providing free map data used by millions of people across the globe.

Registered users can add and edit features to the map in a similar way to Wikipedia.

We added in the legend:

Basemap: OpenStreetMap.

B) REVIEWERS’ MAJOR POINTS

*# 1 REVIEWER: 1. Is the manuscript technically sound, and do the data support the conclusions?
Reviewer #1: Yes*

*Reviewer #2: Partly*

Ok

*# 2 REVIEWER: 3. Have the authors made all data underlying the findings in their manuscript fully available?

The* [*PLOS Data policy*](http://www.plosone.org/static/policies.action#sharing) *requires authors to make all data underlying the findings described in their manuscript fully available without restriction, with rare exception (please refer to the Data Availability Statement in the manuscript PDF file). The data should be provided as part of the manuscript or its supporting information, or deposited to a public repository.*

Ok, revised.

We made the minimal data set related to the values used to build analysis and graphs publicly available without restrictions on the web repository GitHub, accessible at the following link: <https://github.com/cbaldassari/2022-plosone>.

*# 3 REVIEWER: 4. Is the manuscript presented in an intelligible fashion and written in standard English?
Reviewer #1: Yes*

*Reviewer #2: Yes*

Ok

*# 4 REVIEWER: Reviewer #1: This study is more of an exploratory study than a general research paper that reveals causal relationships with research questions. It would be better to express this in the title. It is recommended to mention the general shortcomings and uncertainties of network analysis in relation to this study.*

Ok, revised.

We modified accordingly the title as mentioned above (point # 1 EDITOR).
We introduced and shortly discussed some shortcoming of the network analysis.

Before presenting the results, it is appropriate to point out some disadvantages of using network analysis. These disadvantages apply in general and a fortiori were encountered in the analysis carried out and presented in this article. Firstly, the collection of textual data for network analysis requires careful and meticulous filtering and cleaning to guarantee the accuracy of the terms selected for the construction of graphs between nodes. Secondly, network analysis does not always allow us to propose reliable comparisons between different graphs; in fact, often different networks represent phenomena with different structures and, therefore, not comparable. Consequently, the analysis proposed below will focus on the most macroscopic evidence regarding the structure underlying the different graphs and the most significant patterns.

*# 5 REVIEWER: Reviewer #2:  Their contribution is that authors suggest novel method of industrial classification based on text mining that can be better performed (in what aspect?) comparing with Ateco 2007 or general SIC codes.*

Ok, we gave more emphasis to this point.

Please see below (point # 8 REVIEWER: Reviewer #2)

*\# 6 REVIEWER: Reviewer #2: Overall, although this research suggests the new way of industrial classification, they seek their contribution from the literature on proximity. This mismatch between what they do and what they want to do may mislead readers and make the article lose its coherency.*

Ok, revised.

See below (point # 7 REVIEWER: Reviewer #2).

We followed one of the two reviewer’s suggestions.

*# 7 REVIEWER: Reviewer #2: Since this article is focusing on the classification itself, it would be better to focus on and look for their contribution from the literature on the industrial classification without mentioning proximity measure.*

Ok, revised.

Thank you for this suggestion. It helped us to better circumscribe the literature of reference.

Literature has been revised and the focus of the review has been placed on the studies turned to propose new methods based on alternative techniques to classify firms and industrial activities.

Below the new literature review:

In the modern world, it is crucial to identify correctly and readily innovative firms and classify their industrial activities. However, this information is not easy to elaborate. It is well known that the starting point for understanding what firms do is the SIC system. Using SIC codes is a common practice, even though SIC codes can be uninformative or misleading. The SIC system has many limitations. The descriptions adopted are too concise. The codes, though numerous, are insufficient to represent the variety of existing economic activities (9). The SIC codes are often obsolete and out of step with technological evolution (3). Sometimes, to avoid being ‘confined’ to specific activities or areas, firms opt for broader residual codes. Finally, firms are constantly changing and renewing their businesses: the code chosen yesterday may only partially reflect what the firm does today (10). In addition to the criticalities mentioned above, there is a legitimate question. In the era of big data, in which firms make available a lot of information that can be collected and processed to describe in a detailed and updated way their industrial activities, why not attempt to make some use of it? Many recent contributions propose original methods starting from text data. Below we propose a brief review of the key literature.

(2) developed a sector-product approach and used text mining to enrich the description of firm’s activities in the ICT and digital sector in the United Kingdom. The authors used raw text data and contextual information gathered from websites and news feeds. Interestingly, they affirm that using text mining might provide further detail over SIC codes, which tends to lag far behind technological evolution. (11) proposed the web and new methods of data extraction to derive metadata useful for the industry classification looking at a regional case in the North East of England. The exercise proposed by the authors is a tool to identify specific aggregates of industrial activities in a geographic area. The discussion starts by highlighting the limitations of SIC codes and is followed by the proposed methodology, based on web-based data collection, pre-processing and analysis, and reporting of clusters. (12) conducted a bottom-up study with the aim of overcoming the limitations of industry classifications to study the composition of the economy. Applying machine learning and graph theory techniques, the authors analyse company descriptions extracted from company websites and generate alternative taxonomies on the basis of which they define industries as ‘communities within word networks’. Sometimes the researchers’ intentions do not stop at describing firms’ activities in a new and original way and go further to explore their innovative activity. (13) formulated a novel approach to estimate firms’ innovation activity based on texts on corporate websites. They used automated web-scraper to harvest text from websites, then extracted semantic topics in a self-learning, generative topic-modelling approach, and analyzed these topics using a neural network method to assess each firm’s level of innovation.

Increasingly, existing databases are being used to exploit the huge amount of structured and constantly updated data. They range from large proprietary databases to open repositories: in the former textual data, such as information on the companies’ corporate mission, registered patents and economic and financial news published in the press, are also available, while in the latter, there are descriptive summaries of firms’ activities and products/ services sorted by keywords. (14) analyzed the unstructured texts that describe firms’ businesses using the statistical learning technique of topic modeling and constructed a proximity measure based on the Latent Dirichlet Allocation algorithm, by which represent each firm’s textual description as a probabilistic distribution over a set of underlying topics. (15) performed text mining on Crunchbase to work on green-tech firms in San Francisco, New York and London. Through metadata the authors identified firms’ industrial activities and underlying specializations, building links for technological and market complementarities, identifying specific firms’ aggregates and emerging industrial clusters. (16) measured innovative digital firms’ specializations and competences based on the degree of digital technologies in the products and services supplied. The method allowed to overcome the limitations of defining industrial specializations in digital industries through SIC codes and capture innovative firms’ specializations at the metropolitan level. (17) proposed a classification of specializations along the automotive supply chain in Italy based on the analysis of the descriptive texts of the activities provided in the process of registering with the Chamber of Commerce. The authors implement a multidimensional analysis of words to identify clusters of specializations, and a similarity analysis of words to provide indications on clustering of specializations as they are described by companies.

Sometimes it has been useful to look at alternative information sources than SIC codes to classify economic activities. In this vein are the studies by (8,18) and (19). (8) collected business descriptions from thousands of firm 10-K product descriptions using web crawling algorithms and processed the text to propose new industry classifications. The authors were able to study how firms differ from their competitors using new time-varying measures of product similarity, which allowed to generate a new set of industries in which firms can have their own distinct set of competitors.

However, we have opted to maintain a reference to proximity between firms. In fact, as explained in the text, most of the work aimed at classifying industrial activities also contains a reference to the next step of defining proximity between firms based on the classification obtained.

Consequently, our article also provides matrices of adjacencies based on which firms can be brought closer together because of the similarity of the words used to describe their activities.

At the end of the brief survey of the literature mentioned above, we decided to include a brief mention of the issue of proximity.

Once firms’ activities have been classified, scholars start to address further research questions. One of these concerns the conditions and modalities by which firms exchange knowledge, promoting its diffusion and re-generation. Such an exchange becomes a knowledge flow, something not easily traceable (20). In this case, economists are inclined to use proxies, assuming that these flows have a higher chance to occur between firms that are ‘closer’ in the industry space. In the first place, economists use the SIC system to assume some exchange of knowledge if two firms share the same two-, three-, four-, or five-digit codes (21,22). However, such measures are still discrete, and the level of granularity is constrained by the adopted classification system. Moreover, whether such measures are indicative of firms’ proximity can be questioned (23). The most recent attempts to define and classify industrial activities based on alternative data sources all go hand in hand with the next step of operationalizing proximity between firms. Accordingly, albeit not in depth, this article proposes to explore industrial proximity based on the resulting matrices of adjacencies between firms.

*# 8 REVIEWER: Reviewer #2: If so, author also need to compare the classical way of industrial classification (i.e., SIC) and their text data-driven method of industrial classification to address their contribution to the literature.*

Ok, we gave more emphasis to this point.

The manuscript contains at least three points justifying the use of alternative methodologies and techniques to those based on the SIC codes for classifying the activities carried out by businesses and for assessing their greater informative value.

The opening points are qualitative and are illustrated in the Introduction.

First, this is the reference.

In general, it is not sufficient to refer to the standard industrial classification (SIC) codes to realize firms’ activities and appreciate their degree of innovativeness. SIC codes are a consolidated taxonomy and are currently used for the statistical surveys of economic activities, but they show many limitations as widely discussed in the literature (2,3).

The information attached to the code itself is scarce: this is limited, in fact, to a very short textual definition (e.g., in Europe NACE code M72.1.1 is equivalent to ‘Research and experimental development on biotechnology’). The codes are limited with respect to the existing variety of business activities. Although the codes in the current SIC are more than thousand, they are not (and probably never will be) enough to describe the abundance of firms’ activities: such codes, obsolete and not up to date to technological and market trends, go ‘tight’ to innovative firms. Sometimes firms opt for residual, broader codes such as for example in Europe the NACE code M74.9 – ‘Other professional, scientific and technical activities’: these definitions are too broad and vague to be informative on firms’ activities. Finally, we know that firms innovate, transform, and renew continuously: the code chosen yesterday can only partially reflect what the firm does today or will do tomorrow (4).

Instead of referring to SIC codes, we gather information from firms’ websites and corporate purposes, extract keywords and generate tags concerning firms’ activities, specializations, and competences. Our methodology assumes that firms use the same (or similar) words to identify and describe the same (or similar) activities, regardless of the SIC codes chosen during the registration phase.

The second point goes into the critical issues noted in the literature on the use of SIC codes. This is the starting point for the following short review of the literature.

In the modern world, it is crucial to identify correctly and readily innovative firms and classify their industrial activities. However, this information is not easy to elaborate. It is well known that the starting point for understanding what firms do is the SIC system. Using SIC codes is a common practice, even though SIC codes can be uninformative or misleading. The SIC system has many limitations. The descriptions adopted are too concise. The codes, though numerous, are insufficient to represent the variety of existing economic activities (9). The SIC codes are often obsolete and out of step with technological evolution (3). Sometimes, to avoid being ‘confined’ to specific activities or areas, firms opt for broader residual codes. Finally, firms are constantly changing and renewing their businesses: the code chosen yesterday may only partially reflect what the firm does today (10). In addition to the criticalities mentioned above, there is a legitimate question. In the era of big data, in which firms make available a lot of information that can be collected and processed to describe in a detailed and updated way their industrial activities, why not attempt to make some use of it? Many recent contributions propose original methods starting from text data. Below we propose a brief review of the key literature.

The third point is in the section Results and is the major point:

Is it correct to assume the presence of some links between firms because of the closeness within the Ateco 2007? In our opinion, the approximation is too crude: it is assumed a flow between two firms (only because) near in the SIC system, but nothing can be presumed on the possible content of the flow: what is the content of the exchange? Isolating the content of exchange is very complex, if not impossible, but a deeper investigation of firms’ underlying industrial activities, specializations and competences would allow to deduce something more about the emerging links.

This introduces the analysis based on our proposed approach.

Instead of referring to a SIC system, we gathered information from companies’ websites and corporate purposes and generated tags concerning firms’ activities, specializations, and competences. Using such tags, we built a new dataset, which represented our starting point: the firms observed became sequences of tags that describe their activities or sectors, specializations, and competences.

We modified Figure 3 to give more emphasis to existing differences between the ATECO network and the new one generated from text data.

Please see the point # 12 REVIEWER below.

We added an interpretation of the strongest evidence to capture the advantages of a network based on the proposed methodology (instead of the ATECO hierarchy).

While some clusters of firms are identifiable, such as the blue cluster (ATECO 62) at the bottom right, the green cluster (ATECO 72) above, and the red cluster (ATECO 25) at the bottom left, at the same time it is possible to discern the numerous mixtures between firms belonging to these ATECO codes and firms of different industries. For example, ATECO 25 firms are close in terms of industrial activities to ATECO 22 firms (orange nodes) and to other residual classes (nodes in grey, which refer to ATECO codes with a frequency of less than 3%). ATECO 72 firms at the top (in green) are close to ATECO 70 (light blue), ATECO 74 (light green) and ATECO 64 (violet) firms. The most relevant cluster in terms of nodes (blue, ATECO 62) is divided into three sub-clusters: the bottom right cluster (more colorful and, then, more wide-ranging in terms of ATECO 2-digit codes), the middle cluster with ATECO 73 firms (brown nodes) and close to codes 72 and 74 (dark green and light green, respectively) and, finally, the bottom left cluster (mixed with ATECO 26 firms in bright pink).

C) REVIEWERS’ MINOR POINTS

*# 9 REVIEWER: Reviewer #2: Minor points: 1. Then why author only choose some region instead of entire looking at the entire Italy?*

Ok. As illustrated in the paper, the choice of such geographic area is motivated by the presence of a large and diversified production system. Such a system is well suited to the proposed analysis.

From our point of view, this is the preferred option.

Since we focus on the process of refining the procedural steps and algorithms, we are interested in working on a numerically limited sample so that we could carry out accurate checks and verify the coherence between steps, expected outcomes and attained results.

*\# 10 REVIEWER: Reviewer #2: Minor points: 2. Authors insist that they are looking at “innovative” firms by using keywords, such as digital technology, artificial intelligence, industrial automation, robotics and so on. For me, looking at the entire firms without narrowing their sample sounds more interesting. In addition, authors find that the innovative center is at the sectors “Information and Communication technology” and “Software” based on common specializations. Because they already filtered the keyword, this result is guaranteed from the beginning. If they cover all the firms without filtering using the keywords, their result becomes more meaningful.*

The decision to limit the analysis to such a specific industrial ‘space’ is because new technologies represent a fluid and dynamic area difficult to treat through SIC codes.

The point of the paper is not to select innovative firms based on keywords, but to represent them using text mining and semantic algorithms based on the descriptive text of the firms' activities.

Moreover, circumscribing the industrial activities under observation allows researchers to exploit the depth of the links by capturing the differences and similarities between firms through the co-occurrence of specific keywords.

*# 10 REVIEWER: Reviewer #2: Minor points: 3. In the methodology part, authors choose two taxonomies, which are specialization and competences. Considering that they present their results based on these two taxonomies, more explanation what these two means is required. Please add the explanation and make a reader understood the meaning of the visualized result using the two taxonomies.*

Ok, revised.

We have introduced several considerations aimed at clarifying certain passages.

Regarding the explanation of the graphs, the discussion of the results is limited to what is the most significant evidence, in terms of relevance and novelty/interest on the sample of companies observed.

We used the same colors to compare the networks based on the resulting aggregations of the nodes. See point above # 8 REVIEWER.

*\# 11 REVIEWER: Reviewer #2: Minor points: 4. Figures and tables are not self-complete. Please add more explanation of figures and tables to its caption. For example, what do the colors represent for in figure 2.*

The colors in Figure 2 represent the different sets of firms linked to each other based on the common 2-digit ATECO 2007.

We have included callouts on the figure to allow linking the 2-digit ATECO sectors to identifiable clusters of nodes.

We modified and improved the next figure (Figure 3) to allow comparisons with the network in Figure 2.

*# 12 REVIEWER: Reviewer #2: Minor points: 5. Overall, figures are not reader friendly. For example, hairball like figure 4 should be revised. Please use the way of network visualization in Hidalgo et al. (2009). You can find the supplementary material that describe their visualization method from here:* [*https://www.science.org/doi/10.1126/science.1144581*](https://www.science.org/doi/10.1126/science.1144581)*. Or you can follow the visualization method following Gao et al. (2021). Please see the supporting material of this research as well.*

Ok, revised.

The ‘hairball’ has been modified to be more reader friendly.

Moreover, as suggested in another point, we have modified the Figures 2 and 3 to account for differences between the resulting network based on ATECO and the new one based on text data.

Both figures have the same legend and colors for the ATECO 2-digit codes and this should ease their comparison.

*\# 13 REVIEWER: Reviewer #2: Minor points: 6. The last paragraph of the literature review just put the list of research articles. Please explain the meaning of each literature to utilize it to address your contribution.*

Ok, revised.

The survey of the literature has been consistently revised to account for such and other feedbacks as addressed above (see previous point # 7 REVIEWER).

*# 14 REVIEWER: Reviewer #2: Minor points: 7. Please add page numbers.*

Ok, revised.
